# Role of MYC in B Cell Lymphomagenesis

**DOI:** 10.3390/genes8040115

**Published:** 2017-04-04

**Authors:** Petra Korać, Snježana Dotlić, Maja Matulić, Matea Zajc Petranović, Mara Dominis

**Affiliations:** 1 Department of Biology, Division of Molecular Biology, Faculty of Science, University of Zagreb, Horvatovac 102a, 10000 Zagreb, Croatia; maja.matulic@biol.pmf.hr; 2 Department of Pathology and Cytology, University Hospital Center Zagreb, 10000 Zagreb, Croatia; sdotlic30@gmail.com; 3 Institute for Anthropological Research, Gajeva 32, 10000 Zagreb, Croatia; matea@inantro.hr; 4 Medical School Zagreb, University of Zagreb, 10000 Zagreb, Croatia; mara.dominis@gmail.com

**Keywords:** MYC, B cell, lymphoma, signaling pathways, prognostic factor, meta-analysis

## Abstract

B cell lymphomas mainly arise from different developmental stages of B cells in germinal centers of secondary lymphoid tissue. There are a number of signaling pathways that affect the initiation and development of B cell lymphomagenesis. The functions of several key proteins that represent branching points of signaling networks are changed because of their aberrant expression, degradation, and/or accumulation, and those events determine the fate of the affected B cells. One of the most influential transcription factors, commonly associated with unfavorable prognosis for patients with B cell lymphoma, is nuclear phosphoprotein MYC. During B cell lymphomagenesis, oncogenic MYC variant is deregulated through various mechanisms, such as gene translocation, gene amplification, and epigenetic deregulation of its expression. Owing to alterations of downstream signaling cascades, MYC-overexpressing neoplastic B cells proliferate rapidly, avoid apoptosis, and become unresponsive to most conventional treatments. This review will summarize the roles of MYC in B cell development and oncogenesis, as well as its significance for current B cell lymphoma classification. We compared communication networks within transformed B cells in different lymphomas affected by overexpressed MYC and conducted a meta-analysis concerning the association of MYC with tumor prognosis in different patient populations.

## 1. Introduction

Around 80% of malignant lymphomas belong to the group of mature B cell neoplasms. They arise as a result of the neoplastic transformation of normal B cells in various stages of development and differentiation. Specific B lymphoma types, as a consequence, still demonstrate some, but not all, of the characteristics of normal B cells, reflecting the differentiation stage of a lymphocyte at the time when the oncogenic events occurred [1].

**Figure 1:**
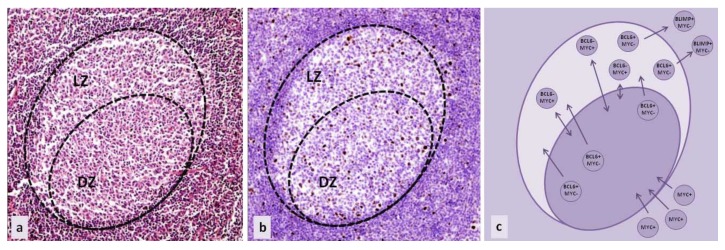
Germinal center of secondary follicle in secondary lymphatic tissue (**a**) haematoxylin and eosin stain (HE), 100×; (**b**) immunohistochemical staining of MYC expressing cells (brown), 100×; (**c**) simplified schematic representation of the regulation of MYC expression in B cell maturation. (DZ—dark zone of germinal center, LZ—light zone of germinal center.)

MYC aberrations were recognized in the majority of aggressive B lymphoma subtypes. In most cases, detected aberrations include the translocations or amplifications of the MYC coding region. The result is MYC overexpression, rather than a change in protein function due to aberrations in the amino acid sequence or protein conformation [1]. Interestingly, B cells in which oncogenic processes take place do not normally express MYC, although their neoplastic counterparts are marked by MYC overexpression. (Figure 1a,b) Furthermore, as a transcription factor, MYC functions both as an activator and a repressor of multiple downstream pathways. Through such regulatory properties, it can promote both proliferation and apoptosis [2,3,4].

It was shown that MYC aberration alone cannot initiate lymphomagenesis. MYC contributes to oncogenic changes and cell transformation, but its aberration is not sufficient for the inducement of tumorigenesis. MYC overexpression adds to the existing oncogenic gene expression profile by enhancing the activity of already active genes in tumor cells [5,6,7]. 

Taken together, these findings suggest that MYC (over)expression in B cell lymphomas is an event that promotes tumor survival and aggressiveness of the disease through a complex interplay of different signaling pathways. Tumor cells start the de novo expression of MYC, and its redundant quantity enables the constitutive activity of already induced oncogenic pathways, in a similar way to how its expression in a subset of normal B cells enhances the already established gene expression profile.

## 2. MYC in B Cell Development and Maturation

B cell development starts in the bone marrow at B lymphocytic progenitor cells. They undergo immunoglobulin VDJ recombination and eventually become mature, naïve B cells that exit the bone marrow, circulate in the peripheral blood, and accumulate in primary lymphoid tissue follicles [1]. These cells are characterized by the expression of surface IgM and/or IgG. After the encounter with specific antigens, their activation is assisted by T cells, triggering the production of transcription factor MYC and the formation of germinal centers (GC). In the germinal centers of secondary follicles, B cells produce transcription factor BCL6, which binds to the promoter of the MYC gene and suppresses its further expression (Figure 1c). This process takes place in the dark zone of the germinal center, which was (until recently) believed to be the zone of highly proliferating centroblasts that undergo somatic hypermutation. The function of BCL6 is to coordinate cell proliferation on one side, and somatic hypermutation and class switch recombination on the other, while inhibiting apoptosis (through targeting of BCL2). BCL6 also regulates different signaling pathways to avoid premature B cell activation and blocks terminal differentiation through repressing BLIMP1, as well as attenuates the DNA damage response, in order to allow further reactions in the germinal center. After the transformation from centroblasts to centrocytes, B cells become part of the light zone of the germinal centers [2,3]. This area was originally believed to be populated by B cells in the later stages of development. However, recently, a different B cell development model was introduced: the light zone only represents a different compartment of the germinal center that is comprised of B cells in a transient state, within the same developmental step as cells in the dark zone. The switching of compartments in the germinal center is important for enabling the high antigen specificity of B cells, and does not represent a change in the differentiation stage [8,9,10,11,12,13]. According to this model, MYC is present in B cells that are acquiring high antigen specificity and ensures the most appropriate antigen affinity. Those B cells are located in the light zone, and can suppress BCL6 expression and re-express MYC [14]. (Figure 1c) After T cell interaction and activation, they re-enter the dark zone of the GC to proliferate and undergo a series of divisions, during which they increase their affinity for specific antigens through additional somatic hypermutation. It was also discovered that the whole program of transition between the GC light and dark zones is regulated by transcription factor E2A or TCF3. TCF3 forms a negative feedback loop with its inhibitor ID3 and their ratio regulates the maintenance of the cell in the dark or light zone of the GC. In the dark zone, TCF3 is preferentially expressed, promoting cell proliferation by inducing cyclin D3 upregulation and downregulating RB1, as well as BCR expression. The upregulation of ID3 decreases TCF3 activity, by forming heterodimers. ID3 expression allows the cells to move to the light zone, and in the light zone, MYC additionally upregulates ID3 [2,3,15,16,17]. 

Other B cells in the light zone of the germinal centers do not re-express MYC—they exit germinal centers, downregulate BCL6 through coordinated activity of several signaling pathways, and express BLIMP1, which further suppresses MYC (Figure 1c). These cells eventually become early plasmablasts or memory cells [2,3].

Regarding the intracellular level, MYC expression can enhance the activation of an already active set of genes, and at the same time, suppress other sets of genes, therefore dysregulating different intracellular cascades. The functional activation of MYC requires heterodimerisation with the transcription factor MAX. The MYC/MAX dimer then binds the CACGTG E-box sequence in the promoter regions of various genes. As a part of that dimer, MYC can regulate the chromatin structure during the gene activation process, by recruiting histone-acetylation complexes to chromatin through the transport protein particle (TRAPP) mediator. In different circumstances, it can modulate the chromatin structure through interaction with other proteins (such as parts of the SWItch/Sucrose Non-Fermentable (SWI/SNF) ATP-dependent chromatin remodeling complex), or it can increase transcription following the recruitment of RNA polymerase II by promoting elongation through the PTEFb transcription factor, and promote RNA polymerase II C-terminal domain phosphorylation and mRNA cap methylation. On the other hand, MYC can suppress almost as many genes as it can activate: in those instances, MYC (as a part of MYC/MAX dimer) will interact with activators already bound to DNA (NFY, MIZ1) and form multiprotein complexes that will be a part of co-activator replacement and co-repressor recruitment [2,3,4,18].

As one of the key players in many different intracellular pathways, MYC can impact cell growth, differentiation, metabolism, angiogenesis, and almost every other process determining the fate of the cell [4]. When its coding sequence is altered in B cell neoplasms, MYC is usually expressed in cells whose normal counterparts do not express it. Moreover, in lymphoma cells, it is overexpressed [1]. All of these functions account for MYC being an important biomarker for various lymphoma subtypes, uniformly as a secondary genetic alteration that usually contributes to an aggressive course of the disease [1,19].

## 3. MYC in B Cell Lymphoma Classification

The last edition of World Health Organization (WHO) Classification of Tumours of Haematopoietic and Lymphoid Tissues was published in 2008, and since then, we have witnessed a significant progress in our understanding of the molecular pathogenesis of lymphomas. This was triggered by the increased availability of modern technologies like next generation sequencing (NGS) or Nanostring, accompanied by large-scale clinical studies that led to new insights into clinical behavior. This prompted a new classification update introduced in 2016, describing major revisions in many lymphoid, histiocytic, and myeloid neoplasms [20].

Over the past few years, MYC rearrangement and activity have been the focus of lymphoma research and clinical trials. Beside Burkitt lymphoma (BL), which was described as an MYC-rearranged lymphoma a long time ago, scientific interest has largely shifted to a heterogeneous family of large B cell lymphomas, and poorly understood aggressive lymphomas composed of medium size and large cells. The latter group has not been precisely defined, so the tumors of such morphology were traditionally categorized together with either ‘true’ diffuse large B cell lymphoma (DLBCL) or BL, or belonged to poorly characterized categories such as “Burkitt-like” lymphomas and B cell lymphoma with features intermediate between DLBCL and BL (BCLU) in previous WHO Classifications. Since the main prerequisites for the optimal management of lymphomas are accurate pathological diagnosis and the provision of predictive biomarkers, MYC became an important point of investigation due to its complex links with a large number of human genes [21].

DLBCL is the most common aggressive type of B cell lymphoma, and approximately 20 years ago, the addition of rituximab to standard chemotherapy regimens (CHOP) resulted in a significant survival improvement of 10–15% [22]. Nevertheless, patients who do not respond to this treatment still die of the disease, which indicates the biological diversity in the family of large B cell lymphomas. DLBCL of the germinal center B cell-like (GCB) phenotype is characterized by the activity of genes which control the cell cycle, apoptosis, and proliferation, such as BCL6 and BCL2. The other group is activated B cell-like (ABC) type DLBCL, in which the NF-κB pathway is activated through permanent stimulation via mutated proteins in the antigen receptor signal transduction [23]. In the updated 2016 WHO classification, new categories of DLBCL and aggressive B cell lymphomas have been introduced, and the category of BCLU has been abolished. 

Recent studies have highlighted the biological and clinical significance of MYC in DLBCL. MYC protein expression can be detected immunohistochemically in 30–50% DLBCL, and is associated with the concomitant expression of BCL2 in 20%–35% of cases [3]. MYC is rearranged in 5%–15% DLBCL, and in a proportion of cases, it is associated with BCL2 or BCL6 translocation. MYC rearrangement, as well as MYC overexpression, affects prognosis, especially in relation to overexpression or co-rearrangement with BCL2. Therefore, DLBCL can be further stratified into types which exhibit neither the co-expression, nor the co-rearrangement of MYC and BCL2, referred to as NOS types, and these confer the best prognosis. Cases where MYC is co-expressed with BCL2, without co-rearrangement, result in intermediate survival and are referred to as “double-expressor” cases, and those with co-rearrangement and the worst prognosis are referred to as “double hit” cases [2,24,25,26,27,28,29,30]. These lymphomas belong to the new category called “High grade B cell lymphoma (HGBL), with rearrangements of MYC and BCL2 and/or BCL6” [20]. Interestingly, lymphomas in this category can morphologically resemble DLBCL, or appear blastoid and Burkitt-like. Morphology is still important for an accurate classification, so B cell lymphomas with blastoid morphology and those morphologically and immunophenotypically intermediate between DLBCL and BL, but without MYC and BCL2/and or BCL6 rearrangement, should be separated from DLBCL and placed in the category of “High grade B cell lymphoma, NOS”. These changes represent the attempt to better define specific lymphoma entities, taking into account the significant overlap in morphology and phenotype, that have for the last few decades, been among the most challenging diagnostic problems for haematopathologists.

Another controversial issue, addressed in the updated 2016 classification, concerns BL, and the question of whether true BL without MYC translocations truly exists. Recent studies identified that 10% of lymphomas that morphologically and phenotypically resemble BL (including pediatric BLs) lack detectable MYC rearrangements, by either fluorescence in situ hybridization (FISH) or classical karyotyping [31,32,33,34]. Instead, a subset of these tumors have a chromosome 11q alteration and a gene expression profile of molecular BL, but a significantly lower MYC expression than classic BL and more complex karyotypes. The clinical course seems to be similar to BL, although the number of cases studied so far is limited. Therefore, it is still uncertain whether these cases represent a variant of BL, and according to the updated WHO classification, they should be diagnosed as a new provisional entity, designated “Burkitt-like lymphoma with 11q aberration” [20].

## 4. Aberrant MYC Signaling Pathways in B Cell Lymphomagenesis

MYC activity is precisely switched on and off in specific steps of B cell differentiation and in specific microenvironments. In concordance with other master regulators, it promotes a series of programs that result in the formation of memory B cells or plasma cells. Each differentiation step can be stopped by oncogenic events that lead to tumorigenesis. Although the deregulation of MYC alone could probably be overridden by cellular “defense” mechanisms, leading to apoptosis of the affected cell, aberrant MYC signaling in lymphomas is accompanied by changes in additional key regulatory pathways that are usually involved in apoptosis. This is called the “double hit” hypothesis and represents one of the processes contributing to an aggressive clinical course in patients with B cell lymphomas [2,35]. 

Most B cell lymphomas appear in lymph nodes, organs responsible for the development of antigen specificity through somatic hypermutation and class-switching mechanisms, which can lead to mutations and translocations of non-Ig genes, if not strictly regulated. These events make B cells especially prone to changes that dysregulate MYC expression and cause mutations in the regulatory sequences of other genes. MYC dysregulation is characteristic of several types of B cell lymphomas. It is believed that in those lymphomas, MYC overexpression arrests normal B cell development and results in cell reprogramming. The phenotype of lymphoma depends on the developmental step in which MYC overexpression occurred and on the specific genetic process that affected its dysregulation. MYC is an example of the oncogene that does not have to change its coding sequence in order to become oncogenic, because unregulated overexpression suffices [36]. Protein overexpression is mainly caused by translocation to highly active chromatin regions. The typical examples are translocations from chromosome 8, where MYC is located, to chromosomes 2, 14, and 22, harboring transcriptionally active loci coding immunoglobulin heavy and light chains, and resulting in the constitutive activity of the MYC promoter [37]. MYC overexpression can also be triggered by DNA amplification or rearrangements involving enhancers, placing them in the vicinity of the MYC promoter. These rearrangements in lymphomas seem to be mediated by activation-induced cytosine deaminase (AID), the driver of genomic rearrangements during normal B cell development [38,39,40]. On the other hand, MYC transcription and prolonged MYC protein stability can be induced by a number of growth factors and signaling pathways. ERK and PI3K pathways cooperate with MYC expression in tumor cells and increase its stability, as well as mutations in certain domains of MYC. It was also demonstrated that the phosphorylation of Ser-62 stabilizes MYC, while Thr-58 phosphorylation leads to MYC degradation. Most of the MYC mutations in lymphomas affect Thr-58, thus changing its phosphorylation and increasing the MYC half-life [41]. Moreover, MYC can be involved in different signaling loops, and its promoter can be constitutively activated by upstream signaling. It can participate in the positive feed-back loop, involving the downregulation of several microRNA (miRNA). In the MYC-miR26a-EZH2-miR494 loop, MYC downregulates miR26a, whose function is to downregulate EZH2, and EZH2 downregulates miR494, which is, in turn, the negative regulator of MYC expression. In this way, the persistent expression of MYC and EZH2 contribute to tumorigenesis [42,43,44].

The WHO Classification of Tumours of Haematopoietic and Lymphoid Tissues published in 2008, included three types of B cell lymphomas where MYC dysregulation was one of the most important aberrations influencing clinical behavior: BL, DLBCL, and BCLU. 

### 4.1. BL

In BL, MYC rearrangements are present in almost all of the cases. The karyotype is simple, which means that very few other aberrations are present [36]. That is why BL became the classical model for investigation of oncogene activation by chromosomal translocation. Translocation from chromosome 8 to chromosomes carrying Ig genes leads to the constitutive activity of the MYC promoter. In BL, there is a higher incidence of MYC translocations to Ig heavy chain loci, in comparison to other types of lymphomas where MYC is translocated to Ig light chain loci, or to other non-Ig loci. Furthermore, upstream regulators of B cell maturation, the TCF3 and ID3 genes, are often mutated in cells carrying MYC translocation [45]. MYC genes are usually translocated to highly transcriptionally active regions, usually involved in Ig chain production. These translocations are induced by AID, an enzyme which enables DNA mutations important for antibody affinity maturation [46,47]. Its function involves the creation of nicks and double-strand breaks during the process of class switch recombination and somatic hypermutation in cells of germinal centers in the lymph nodes. Additional oncogenic aberrations in BL might be associated with different pathogenetic mechanisms involved in BL subtypes. In the endemic Burkitt lymphoma, occuring in central Africa, the disease is often associated with coincident Epstein-Barr virus (EBV) and malaria infection. In those cases, AID is probably upregulated in the germinal centers of secondary lymphoid tissue as a consequence of *Plasmodium* infection, while EBV contributes to lymphomagenesis by increasing pro-survival signaling [48]. In this BL subtype, MYC is translocated to non-heavy chain immunoglobulin loci, as a side effect of the somatic hypermutation process which generates DNA breaks. In the immunodeficiency-associated form of BL, tumor development is associated with EBV or human immunodeficiency virus (HIV) infection and a third form of this disease is immunosuppression-related. In those cases, MYC is translocated to the immunoglobulin heavy chain locus [18]. 

All subtypes of BL are probably derived from the germinal center dark zone cells. In this zone, ectopic MYC overexpression alone could lead to apoptosis: an increase in E2F can upregulate the p53 pathway and lead to cell death as part of a cell defense mechanism. In BL, apoptosis is prevented through additional aberrations involving some of the other key regulators mentioned above, or through the activation of the PI3K signaling pathway. Nearly 70% of BL also bear mutations in upstream regulators involved in the TCF3-ID3 pathway, leading to increased cell survival. Thus, it seems that TCF3 can promote survival through BCR signaling, independent of the antigen, and the activation of PI3K signaling could be a downstream consequence of TCF3 dysregulation [49]. In parallel, MYC can influence ID3-TCF3 regulation and therefore influence cyclin D3 expression, as well as increase proliferation and cell growth [45,50].

### 4.2. DLBCL

In a subset of DLBCL (DLBCL, not otherwise specified), the cell of origin can be a germinal center B cell from either the light or dark GC zone. As previously mentioned, based on the gene expression profile of the cell of origin, DLBCL was divided into two main subgroups: GCB and ABC subtypes [1]. MYC overexpression is typical for the aggressive type of lymphoma with the GCB phenotype, in which it cooperates with other factors influencing signaling cascades that contribute to the process of lymphomagenesis. There are many known mutations in DLBCL, but the most important ones include those affecting the genes involved in epigenetic modifications (such as mutations in acetyltransferases and histone methyltransferase MLL2), as well as those involved in the regulation of proliferation, differentiation, and apoptosis, such as BCL6 and BCL2. Chromatin modifiers also influence the expression of a number of genes, such as p53 and BCL6 proto-oncogenes. The BCL6 locus is often involved in chromosomal translocations, placing BCL6 near the IGH locus or near other highly activated promoters. BCL6 dysregulation can be found in nearly 30% of DLBCL cases, where it affects the autoregulatory loop or selection of promoter regions involved in its repression [1]. Furthermore, BCL6 dysregulation abrogates the process of B cell differentiation once the lymphocytes exit germinal centers, as well as apoptosis. The direct function of BCL6 in lymphomagenesis is still a subject of investigation, although its regulatory role in coordinating processes in the germinal center has been thoroughly studied so far. It is possible that the induction of persistent tolerance to DNA damage leads to the accumulation of oncogenic mutations, such as MYC translocations [51,52,53,54,55]. In turn, the constitutive expression of MYC results in the abrogation of its BCL6-mediated transcriptional repression, normally present in the dark zone of the germinal center. Additionally, sets of genes affected by translocations and other activating/inactivating mutations in GCB-DLBCL are linked together in signaling circuits (chromatin remodelers, cyclin dependent kinases, BCL6, BLIMP1, MYC and BCL2), leading to an increase in proliferation and escape from apoptosis. Also, in DLBCL, the signaling involving cell migration and survival pathways is often dysregulated, enabling neoplastic cells to leave the lymph node and enter the circulation, thus escaping normal controlling mechanisms [16,51,56,57,58,59]. In addition, in approximately one quarter of ABC-DLBCL cases, BLIMP1 is disrupted by inactivating mutations [59]. 

### 4.3. BCLU

The WHO classification from 2008 included the provisional category of BCLU as a heterogeneous group of lymphomas showing morphologic and immunophenotypic features which are similar to both typical DLBCL and BL. MYC rearrangements were found in 35%–50% of all BCLU cases. They were frequently accompanied by BCL2 and BCL6 translocations as a part of complex karyotype, and in some cases, present as a “double hit” or “triple hit” lymphoma [18]. A second genetic lesion usually involved genes engaged in mechanisms controlling escape from apoptosis, thus leading to cell survival. MYC/BCL2 lymphomas were described as neoplasms with a phenotype characteristic of germinal center B cells: they express BCL6 and CD10, and lack IRF4, a regulator of BCL6 [60]. 

### 4.4. Other B Cell Lymphomas

Besides having a crucial impact on complex signaling networks in aggressive lymphomas, MYC is also associated with other B cell and plasmacytic neoplasms. MYC rearrangement or dysregulation occurs as a secondary change in their tumorigenesis, leading to a more aggressive type of the disease in a subset of patients. 

MYC activation can be found in nearly 50% of plasmablastic lymphomas (PBL), a rare, aggressive B cell lymphoma, usually occurring in patients with immunodeficiency or immunosuppression related to ageing, therapy, or the presence of EBV infection. Plasmablastic lymphoma is characterized by the phenotype of terminally differentiated B cells, without the expression of mature B cell- or plasma cell-related markers. Plasma cell myeloma (PCM) is a neoplasm composed of monoclonal plasma cells. In both tumors originating from B cells in terminal stages of differentiation, MYC aberrations are found in more aggressive forms of the disease. Interestingly, gene expression array analysis showed that a MYC activation signature is detected in plasma cells in patients with myeloma, but not in monoclonal gammopathy of undetermined significance (MGUS), which precedes PCM in most patients [61]. In PBL, the MYC locus is usually translocated to the immunoglobulin gene, without rearrangements in BCL6 and BCL2 loci. In PCM, MYC is commonly translocated to non-Ig loci. PBL cells express BLIMP1, the master regulator of B cell differentiation. BLIMP1 directs the differentiation toward plasma cells, promoting the expression of specific plasma cell markers and suppressing those of mature B cells. As mentioned above, BLIMP1 in normal cells suppresses MYC and controls genes involved in proliferation. In neoplastic plasma cells, MYC overexpression and activation of the unfolded protein response (UPR), which induces the expression of antiapoptotic genes, enables MYC to avoid repression by BLIMP1, thus allowing neoplastic cells to avoid apoptosis [36,62]. 

Anaplastic lymphoma kinase (ALK)-positive large B cell lymphoma is an aggressive neoplasm composed of cells with a plasmablastic phenotype, expressing the ALK protein. The pattern of ALK expression depends on specific partner genes involved in the translocation. These tumors, similar to plasmablastic lymphoma, express BLIMP1 and plasma cell markers, without the expression of mature B cell antigens. MYC expression in ALK+ lymphoblastic lymphoma (LBL) is upregulated, probably by the STAT3 dependent activation of the MYC promoter. STAT3 is, on the other side, phosphorylated by ALK. STAT3 also induces the expression of BLIMP1 and plasma cell differentiation, but MYC activation abrogates BLIMP1-mediated regulation. It is possible that, similar to PBL, the activation of the UPR, in addition to MYC expression, enables neoplastic cells to survive and proliferate [2,62,63]. 

MYC dysregulation also occurs in other types of B cell neoplasms, in which its expression leads to disease progression and the transformation to more aggressive forms. Follicular lymphoma is usually an indolent disease, characterized by BCL2 translocation, abrogating cell apoptosis in the germinal center. By obtaining MYC translocation in addition to BCL2 rearrangement, these cells transform to cells that morphologically and phenotypically resemble Burkitt lymphoma or DLBCL, and these patients invariably have a poor prognosis [64]. Mantle cell lymphoma is a mature B cell lymphoma with characteristic IGH/CCND1 translocation and/or overexpression of cyclin D1. A number of cases of this aggressive lymphoma (usually pleomorphic/blastoid variant of MCL) are associated with MYC overexpression, often through MYC translocation to an additional IGH locus [65]. Chronic lymphocytic leukemia/small lymphocytic lymphoma (CLL/SLL) is characterized by small lymphocytes that are derived from mature, antigen experienced B cells and usually show a CD19^+^CD5^+^CD23^+^ immunophenotype. The course of the disease can be variable and many prognostic markers have been described so far. In rare cases, CLL can transform to aggressive lymphoma (usually DLBCL)—the phenomenon called Richter syndrome (RS). In most RS cases, MYC aberrations were found, but it is still not clear whether MYC aberration is the trigger of disease progression or one of the events contributing to the already induced progression. Nevertheless, the association of MYC translocation with an unfavorable prognosis of CLL/Richter syndrome patients was confirmed in several studies [1,66,67].

In B lymphoblastic lymphoma/leukemia, the occasional occurrence of MYC aberrations triggers the more aggressive form of the disease [68].

In addition to all described mechanisms of lymphomagenesis in which MYC drives cell proliferation and survival, God et al. (2015) found that MYC overexpression in B cell lymphomas decreases the immunogenicity of the neoplasm by decreasing HLA Class II-mediated immune recognition [69].

MYC overexpression in tumor cells, regardless of the underlying mechanism, influences many different signaling pathways, all resulting in **a** more aggressive clinical course of the disease.

## 5. MYC Overexpression as a Prognostic Marker

Although MYC expression is associated with tumorigenesis in almost all B cell lymphomas that arise from germinal center cells, most authors identified it as one of the key prognostic and predictive biomarkers for survival in DLBCL, and some showed that MYC overexpression is associated with the worst survival rates [25,30,70,71,72]. Still, during the examination of the PubMed database, we found only one meta-analysis which tested the association of c-MYC protein expression and the overall survival (OS) in four individual studies: the overall pooled hazard ratio (HR) estimate was 2.13 (95% CI, 1.55–2.91) [73]. To investigate this finding further, we performed a systematic review and meta-analysis of 15 studies, including 3001 patients in total [25,70,72,73,74,75,76,77,78,79,80,81,82,83,84]. The meta-analysis was performed according to PRISMA guidelines and the detailed description of the methods and data is presented in the Supplementary Material [85].

Nine studies composed of 2265 patients, 30.8% of whom displayed high MYC expression, reported data on adjusted estimates of OS (Table 1). 

**Figure 2:**
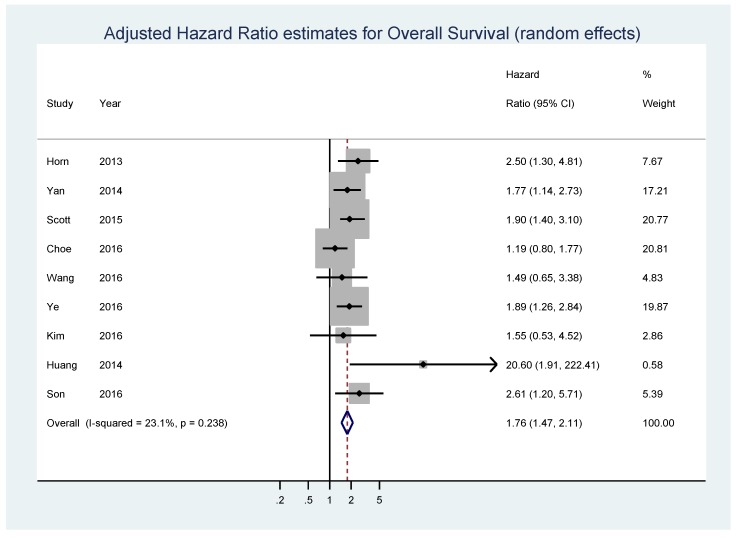
Forest plot showing the meta-analysis of adjusted hazard ratio estimates for overall survival (OS) in patients with MYC overexpression.

The pooled analysis showed that high MYC expression was associated with shorter OS (HR = 1.76, 95% CI = 1.47–2.11, *p* < 0.001; I_2_ = 23.1%, *p* = 0.238 for heterogeneity) (Figure 2). No significant bias was found by the Begg’s test (*p* = 0.466) and Egger’s test (*p* = 0.104) (Figure S2).

**Figure 3:**
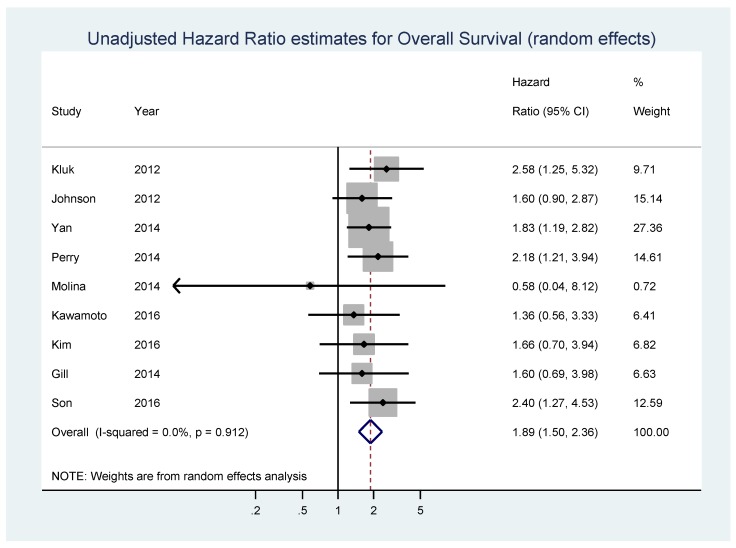
Forest plot showing the meta-analysis of unadjusted hazard ratio estimates for overall survival (OS) in patients with MYC overexpression.

Nine studies composed of 1127 patients, 42.1% of whom displayed high MYC expression, reported data on unadjusted estimates of OS (Table 1). In the pooled analysis of unadjusted HR estimates of OS, a statistically significant association between MYC overexpression and shorter OS values was found (HR = 1.89, 95% CI 1.50–2.36, *p* < 0.001; I_2_ = 0%, *p* = 0.912 for heterogeneity) (Figure 3). No significant bias was found by the Begg’s test (*p* = 0.175) and Egger’s test (*p* = 0.215) (Figure S3).

These results indicate that high MYC expression is indeed significantly associated with shorter OS values (both adjusted and unadjusted for covariates) in patients with diffuse large B cell lymphoma. Bearing in mind that diffuse large B cell lymphoma is the most frequent lymphoma associated with the germinal center cell of origin, and that limitations of this data (included studies differed in study design, applied cut-offs for MYC overexpression and chemotherapy treatment) did not contribute to high or statistically significant heterogeneity, MYC (over)expression can be regarded as a potential target for precise therapy in MYC-expressed lymphoma entities.

## 6. MYC-Based Therapy and Future Perspective

Currently, there are few MYC-based therapeutic approaches. There are two target functions of MYC once it is already expressed in a cell: dimerization with MAX and MYC/MAX binding to DNA. The target of another approach is the epigenetic regulation of MYC expression through the inhibition of BRD4, and in other approaches, of downstream members of MYC-induced cascades in neoplastic cells.

MYC must dimerize with MAX through basic helix-loop-helix leucine zipper protein domains (bHLH-Zip), in order to bind chromatin and enhance the transcription of downstream genes [86]. This coupling of proteins is based on changes in the configuration of a binding domain specific for different protein partners. In the case of MYC, it suggests that the disruption of the MYC/MAX binding site can be a strategy for the inactivation of MYC function in neoplastic cells. Such an approach was already applied and different small molecule inhibitors that can specifically target MYC were already successfully produced [87,88,89,90,91,92,93,94,95].

The second approach is based on the inhibition of MYC/MAX dimers binding to E-boxes in the promoters of different genes whose transcriptions are enhanced. MYC pathway response agents (MYRAs) were thus constructed in order to prevent association between protein dimers and DNA. Those compounds were able to induce a high apoptotic rate in lymphoma cells [96]. 

BRD4 belongs to the bromodomain and extra-terminal (BET) family of proteins. Its bromodomain binds with high affinity to acetylated histones in enhancer sequences. When bound to chromatin, BRD4 recruits transcription effectors and activates P-TEFb, which consequently promotes MYC expression [97,98,99,100,101,102]. In order to interfere with BRD4 and its binding sites in chromatin, small-molecule compounds were constructed. Some of them, such as JQ1, I-BET762, OTX015, and I-BET151 reduced the MYC expression caused by translocation or amplification, or had an antiproliferative effect dependent on the downregulation of MYC-targeted genes [99,103,104,105,106].

Yet another possible approach to MYC-based tumorigenesis is to induce synthetic lethality. This is a process that represents the co-occurrence of two genetic events that result in cell death. In MYC overexpression, this means that the targets are several signaling pathways required for the neoplastic cell function, which are not essential in normal B cells. Inhibitors of certain proteins in downstream cascades were produced that can cause neoplastic, but not normal, B cell death [107,108].

All treatment designs mentioned above are based on transcriptional or protein function levels, rather than a gene-based approach. This is due to the fact that MYC has important roles in both normal and tumorigenic B cells, and its deletion caused embryonic lethality in mice because of developmental defects in multiple organs [109].

However, new tools for (epi)genetic editing emerge that allow for highly specific genome manipulation. Precise genome editing started with zinc-finger nucleases (ZNF), became easier and increasingly used with transcription activator-like effector nucleases (TALEN), and was finally widespread due to rapid, simple, and flexible clustered regularly interspaced short palindromic repeat (CRISPR) sequence and CRISPR-associated (Cas) proteins, that were proved to work in mammalian cells with great efficiency [110,111,112,113]. These approaches are based on inducing targeted DNA damage and subsequent repair, which results in permanent changes of the genomic sequence. Cas9 nuclease is guided to the specific genomic site by a single guide RNA (gRNA), which contains a 20 bp recognition sequence specific for the targeted DNA. A nuclease-induced break is repaired through an error-prone mechanism, often resulting in mutagenesis and gene knockout. The same approach can also be used for the directed insertion of sequences in a process of sequence replacement, using homologous recombination. Other options are the replacement of the Cas9 nuclease function by another enzyme, so that gRNA can specifically modify not only the DNA molecule, but also, for example, the chromatin structure [114,115,116,117]. In this way, MYC can be targeted on different levels. After the confirmation of genetic change in neoplastic B cells, MYC overexpression caused by translocation can be targeted via a translocation partner [118]. In those scenarios, MYC knock-out would be restricted to the aberrant MYC copy in the cell. Furthermore, if constructed in a way that gRNA brings about methyltransferase, histone deacetylase, or some other epigenetically important enzyme, it could silence oncogenic MYC copies in tumor cells. Similar approaches were already tested in different studies on different models, and their results promise new, efficient mechanisms for the precise inactivation of MYC. Although MYC overexpression is most likely a secondary event in B cell lymphomagenesis, its association with disease aggressiveness makes it a unique and favorable candidate for such therapeutic approaches.

## Figures and Tables

**Table 1:** Baseline characteristics of eligible studies.

Study	Year	Population	Disease	Number of Patients	Detection Method	Available Data	Median, Follow-Up, Months (Minimum,Maximum)	Threshold for MYC Expression	N myc_high	N myc_low	Origin of Data
Kluk [69]	2012	USA (Boston, Massachusetts)	diffuse large B-cell lymphoma	38	IHC	OS-uni	42 (2,87)	>50%	6	32	extrapolated
Johnson [25]	2012	not specified (from 10 international institutions)	de novo diagnosed diffuse large B-cell lymphoma	307	IHC/Microarray	OS-uni	49 (6,136)	≥40%	100	207	reported in text
Horn [70]	2013	Germany (samples taken from the RICOVER study of the German High-Grade Non-Hodgkin Lymphoma Study Group (DSHNHL)	diffuse large B-cell lymphoma	283	IHC/FISH	OS-multi, EFS-multi	29 (4,64)	≥40%	43	98	reported in text
Yan [71]	2014	China	diffuse large B-cell lymphoma	203	IHC	OS-uni, PFS-uni; OS-multi, PFS-multi	37 (1,145)	≥40%	108	95	reported in text
Perry [67]	2014	USA (patients from the Nebraska Lymphoma Study Group cohort)	diffuse large B-cell lymphoma	106	IHC	OS-uni	54 (7,145)	>50%	69	37	extrapolated
Molina [72]	2014	France (participants in the Groupe d’Etudes des Lymphomes de l’Adulte/Lymphoma Study Association (LYSA) LNH 03-2B trial)	previously untreated de novo CD20+ difuse large B-cell lymphoma	174	IHC	OS-uni, PFS-uni	not specified	≥40%	47	127	reported in text
Huang [73]	2014	China (samples collected at the Cancer Institute and Hospital, Chinese Academy of Medical Sciences (CICAMS) in Beijing)	MALT lymphoma without LTCs patients, 20 cases of MALT lymphoma with LTCs and 7 cases of DLBCL with a MALT lymphoma component	62	IHC	OS-multi	43 (1,84)	≥20%	16	46	reported in text
Gill [74]	2014	USA	primary diffuse large B-cell lymphoma of the central nervous system (CNS DLBCL)	59	IHC	OS-uni	22 (<1,128)	≥40%	43	16	calculated from raw data in supplemental table
Scott [75]	2015	Pretreatment tumor biopsies of patients diagnosed with de novo DLBCL, treated at the British Columbia Cancer Agency	de novo untreated diffuse large B-cell lymphoma	339	IHC	OS-multi, PFS-multi	78 (9,158)	≥40%	105	234	reported in text
Choe [76]	2016	Korea	diffuse large B-cell lymphoma	173	IHC	OS-multi	not specified	≥20%	not specified	not specified	reported in text
Wang [77]	2016	USA (Nashville, Tennessee and Houston, Texas)	de novo untreated diffuse large B-cell lymphoma	192	IHC	OS-multi	not specified	≥40%	106	86	reported in text
Kawamoto [78]	2016	Japan (Nigata)	diffuse large B-cell lymphoma	52	IHC	OS-uni, PFS	76 (4,127)	≥30%	32	29	reported in text
Ye [79]	2016	USA (participants in the The International DLBCL Rituximab-CHOP Consortium Program Study)	de novo untreated diffuse large B-cell lymphoma	825	IHC	OS-multi	59 (1,187)	≥70%	249	576	reported in text
Kim [80]	2016	Korea (patients from the Seoul National University Hospital)	primary diffuse large B-cell lymphoma of the central nervous system	114	IHC	OS-uni, PFS-uni; OS-multi, PFS-multi	83 (0.2,118)	≥40%	21	93	calculated from raw data in supplemental table
Son [81]	2016	Korea (Department of Pathology of the Samsung Medical Center in Seoul)	pretreatment tumor biopsies of patients diagnosed with de novo central nervous system diffuse large B-cell lymphoma	74	IHC	OS-uni ; OS-multi	35.2 (1.8,148.8)	≥44%	49	25	reported in text

OS, overall survival; EFS, event free survival; PFS, progression free survival; IHC, immunohistochemistry; FISH, fluorescent in situ hybridization.

## Supplementary Materials

The following are available online at www.mdpi.com/2073-4425/8/4/115/s1, Supplementary Material: Methods, Search strategy, Data extraction, Statistical analysis, Eligible studies, Overall survival, Figure S1: A flowchart of a selection of studies eligible for the MYC overexpression meta-analysis, Figure S2: Begg’s funnel plot for adjusted estimates in overall survival (OS) meta-analysis, Figure S3: Egger’s funnel plot for adjusted estimates in overall survival (OS) meta-analysis, Figure S4: Influential meta-analysis plot for adjusted effect estimates in overall survival (OS), after omitting an individual study each time, Figure S5: Begg’s funnel plot for unadjusted estimates in overall survival (OS) meta-analysis, Figure S6: Egger’s funnel plot for unadjusted estimates in overall survival (OS) meta-analysis, Figure S7: Influential meta-analysis plot for unadjusted effect estimates in overall survival (OS), after omitting an individual study each time, Figure S8: Forest plot showing the meta-analysis of adjusted hazard ratio estimates for overall survival (OS) in patients with MYC overexpression defined as MYC ≥ 40%.
