# Supplementary material for: Role of MYC in B Cell Lymphomagenesis
Source: Genes (Basel). 2017 Apr 4;8(4):115. doi: 10.3390/genes8040115 (PMC5406862; doi:10.3390/genes8040115)
Supplement: Supplementary file 1 [file genes-08-00115-s001.docx]

**Supplementary Material**

Methods

The present meta-analysis was performed according to PRISMA guidelines [85].

Search strategy

The PubMed database was searched until December 1^st^, 2016. The keywords used in the online search were: “B-cell lymphoma”, “MYC overexpression”, and “overall survival”. Only full-text papers written in English were included. The eligibility criteria were: 1) human-based studies; 2) diagnosis of any type of B-cell lymphoma; 3) defined cut-offs for MYC overexpression; 4) adjusted and/or unadjusted hazard ratio (HR) estimates for Overall Survival (OS) stratified by MYC overexpression; and 5) in case criteria 4) was not satisfied, studies which included raw data from which HR could be calculated were included.

Data extraction

The following data were extracted from the selected studies: the first author’s name, year of publication, country where the study was conducted, a brief description of patients from whom samples were taken, disease, sample size, detection method of MYC overexpression, median follow-up (in months), threshold for MYC high/low expression, number of MYC high expression patients, number of MYC low expression patients, and covariates utilized in univariate and/or multivariate analyses of OS.

Statistical analysis

The Hazard Ratios (HRs) and 95% Confidence Intervals (CIs) were employed to evaluate the relationship between MYC overexpression and OS. Data were combined using a fixed effects method. Heterogeneity between studies was assessed by the I^2^ statistics. Publication bias was tested by Beggs’s and Egger’s tests. All statistical analyses were carried out using STATA version 17 (Stata Corporation, College Station, TX, USA).

Eligible studies

In the initial search, seventy-four potentially relevant articles were identified using keywords (Figure S1). Six were excluded because these were review papers and 25 were excluded because the reported data were on non-humans: 43 full-text articles were assessed for eligibility. Among them, a further four articles were excluded because they were duplicates, and another 24 because the reported data were insufficient or the topic of the paper did not fit our study design. At the end, 15 studies containing 3001 patients were included in the meta-analysis.

The baseline characteristics of the included studies are as follows (in details presented in Table 1): studies were published between 2012 and 2016 and sample sizes of datasets ranged from 38 to 825. The ethnicity of the patients in most of the studies was not stated clearly. In two studies, MYC overexpression was defined as MYC ≥ 20%, in one study as MYC ≥ 30%, in eight studies as MYC≥40%, in one study as MYC ≥ 44%, in two as MYC > 50%, and in one as MYC ≥ 70% [25,70,72–83].

Overall survival

Nine studies composed of 2265 patients, of whom 697 (30.8%) displayed high MYC expression, reported data on adjusted estimates for OS (Table 1). The covariates reported in the included studies were: gender in all studies; the International Prognostic Index (IPI; ie, age > 60, lactate dehydrogenase [LDH] > normal, Eastern Cooperative Oncology Group (ECOG) > 1, stage III/IV, and extranodal involvement > 1) and rituximab treatment; IPI, immunohistochemical subgroups (GCB vs. non-GCB), MYC, BCL2, and BCL6 expression, as well as the cyclophosphamide, doxorubicin, vincristine, and prednisone treatment (CHOP); BCL2 protein; age, Ann Arbor stage, ECOG score, treatment and PELI1(>2); MYC-rearrangement, P53 expression, and IPI; IPI, B-symptoms, tumor size, MYC, BCL2 and BCL6 translocation status, BCL2 and BCL6 expression; Nottingham-Barcelona score and (Memorial Sloan-Kettering Cancer Center prognostic model (MSKCC); IPI, LDH and Ann Arbor stage; and ECOG status, cell-of-origin phenotype according to the Hans algorithm, BCL6 and BCL2 protein expression, and MYC mRNA expression status [73,75,763,78–80, 82–84]. The sensitivity analysis did not indicate that any individual study significantly affected the pooled HR for OS (Figure S2-S4).

A further nine studies composed of 1127 patients, of whom 475 (42.1%) exhibted high MYC expression, reported data on unadjusted estimates for OS (Table 1). The sensitivity analysis of eight studies (Molina et al. 2014 had to be excluded because ln<0) did not indicate that any individual study significantly affected the pooled HR for OS (Figure S5-S7) [75].

In order to eliminate a possible influence of different MYC overexpression cutoffs on the HR estimates for OS, we performed an additional pooled analysis of adjusted HR estimates on studies with MYC ≥ 40% (most widely accepted cutoff): a statistically significant association between MYC overexpression and shorter OS was found (HR = 1.92, 95% CI 1.56 – 2.35, *p* < 0.0001; I^2^ = 0%, *p* = 0.930 for heterogeneity) (Figure S8).

Records identified through Pubmed database searching

(*n* = 74)

## Identification

Records after reviews removed
(*n* = 68)

## Screening

Records excluded for non-humans (*n* = 25)
(n = )

Records screened
(*n* = 43)

## Eligibility

Full-text articles excluded for incompatibility with topic or insufficient data
(*n* = 24)

Full-text articles assessed for eligibility
(*n* = 39)

## Included

Studies included in

meta-analysis
(*n* = 15)

**Figure S1.** A flowchart of a selection of studies eligible for the MYC overexpression meta –analysis.

*p* = 0.466

**Figure S2.** Begg`s funnel plot for adjusted estimates in overall survival (OS) meta –analysis.

*p* = 0.104

**Figure S3.** Egger`s funnel plot for adjusted estimates in overall survival (OS) meta –analysis.

3.15

4.26

3.27

5.55

9.58

1

2

3

4

5

6

7

8

9

Study ommited

Meta-analysis fixed-effects estimates (exponential form)

**Figure S4.** Influential meta –analysis plot for adjusted effect estimates in overall survival (OS), after omitting an individual study each time.

*p* = 0.175

**Figure S5**. Begg`s funnel plot for unadjusted estimates in overall survival (OS) meta –analysis.

*p* = 0.215

**Figure S6.** Egger`s funnel plot for unadjusted estimates in overall survival (OS) meta –analysis.

3.48

5.19

3.64

7.41

9.40

1

2

3

4

5

6

7

8

Study ommited

Meta-analysis fixed-effects estimates (exponential form)

**Figure S7.** Influential meta –analysis plot for unadjusted effect estimates in overall survival (OS), after omitting an individual study each time.

**Figure S8.** Forest plot showing the meta-analysis of adjusted hazard ratio estimates for overall survival (OS) in patients with MYC overexpression defined as MYC ≥ 40%.
